# Identification of residues required for stalled-ribosome rescue in the codon-independent release factor YaeJ

**DOI:** 10.1093/nar/gkt1280

**Published:** 2013-12-09

**Authors:** Hiroyuki Kogure, Yoshihiro Handa, Masahiro Nagata, Naoto Kanai, Peter Güntert, Kenji Kubota, Nobukazu Nameki

**Affiliations:** ^1^Division of Molecular Science, Faculty of Science and Technology, Gunma University, 1-5-1 Tenjin-cho, Kiryu, Gunma 376-8515, Japan, ^2^Institute of Biophysical Chemistry, Center for Biomolecular Magnetic Resonance, and Frankfurt Institute for Advanced Studies, Goethe University, Frankfurt am Main, Germany and ^3^Graduate School of Science and Engineering, Tokyo Metropolitan University, 1-1 Minami-ohsawa, Hachioji, Tokyo 192-0397, Japan

## Abstract

The YaeJ protein is a codon-independent release factor with peptidyl-tRNA hydrolysis (PTH) activity, and functions as a stalled-ribosome rescue factor in *Escherichia coli*. To identify residues required for YaeJ function, we performed mutational analysis for *in vitro* PTH activity towards rescue of ribosomes stalled on a non-stop mRNA, and for ribosome-binding efficiency. We focused on residues conserved among bacterial YaeJ proteins. Additionally, we determined the solution structure of the GGQ domain of YaeJ from *E. coli* using nuclear magnetic resonance spectroscopy. YaeJ and a human homolog, ICT1, had similar levels of PTH activity, despite various differences in sequence and structure. While no YaeJ-specific residues important for PTH activity occur in the structured GGQ domain, Arg118, Leu119, Lys122, Lys129 and Arg132 in the following C-terminal extension were required for PTH activity. All of these residues are completely conserved among bacteria. The equivalent residues were also found in the C-terminal extension of ICT1, allowing an appropriate sequence alignment between YaeJ and ICT1 proteins from various species. Single amino acid substitutions for each of these residues significantly decreased ribosome-binding efficiency. These biochemical findings provide clues to understanding how YaeJ enters the A-site of stalled ribosomes.

## INTRODUCTION

Ribosomes often stall when translating irregular mRNAs, such as a non-stop mRNA generated by abortive transcription by RNA polymerase, or RNA digestion by RNase (1,2). These stalled ribosomes would be deleterious for cells if left uncorrected, therefore all bacteria have at least one system to release such stalled ribosomes. The *trans*-translation system is mediated by transfer-messenger RNA (tmRNA) associated with SmpB (3–5). This system contains a mechanism of incomplete protein degradation, in which a tag peptide encoded by tmRNA is added to the C-terminus of the growing polypeptide, and the resulting tagged protein is immediately degraded by several tag-specific proteases. This process consequently promotes ribosome recycling and truncated mRNA degradation (6), and prevents accumulation of potentially deleterious truncated polypeptides during normal cell growth (7,8).

Recent *in vivo* and *in vitro* experiments using *E. coli* have revealed two other stalled ribosome rescue systems. Synthetic lethality screening experiments showed that the *yhdL* gene product (ArfA) is essential for the viability of *E. coli* in the absence of tmRNA (9). ArfA can rescue stalled ribosomes in a tmRNA-independent manner, but cannot hydrolyze peptidyl-tRNA from nonstop mRNA by itself (10). Instead, a class I polypeptide chain release factor (RF) 2, collaborates with ArfA to release nascent chains. It is noteworthy that the ArfA rescue system is a back-up to the tmRNA rescue system because functional ArfA is poorly expressed under conditions where the tmRNA system is in operation (11). Whereas the tmRNA system is ubiquitous among bacteria, the alternative rescue pathway mediated by ArfA is narrowly distributed amongst a subset of β- and γ-Proteobacteria (12).

The other stalled-ribosome rescue factor is the YaeJ protein, which contains a Gly-Gly-Gln (GGQ) motif that is invariably conserved in the catalytic domain (domain 3) of the class I polypeptide chain RFs. YaeJ homologs have been identified in many Gram-negative bacteria, with some exceptions such as *Thermus thermophilus*, but not in Gram-positive bacteria (13). *In vitro* translation experiments using the *E. coli*-based reconstituted cell-free protein synthesis system have revealed that YaeJ can hydrolyze peptidyl-tRNA on ribosomes stalled on not only non-stop mRNAs but also mRNAs containing a cluster of rare codons that extend downstream from the P-site (13). This hydrolysis is achieved by using the GGQ motif residues as a catalytic site in the same manner as RF, and thus YaeJ can be termed a codon-independent RF (ciRF). In addition, sucrose density gradient centrifugation analysis has shown that YaeJ is more commonly associated with 70S ribosomes than with the 50S or 30S subunits.

Synthetic lethality screening experiments demonstrated that the lethal phenotype of an *ssrA* (the tmRNA gene) and *arfA* (*yhdL*) double mutant is suppressed by overexpression of plasmid-encoded YaeJ, but not by the products derived from a genomic copy of *yaeJ* (14). Thus, it is most likely that YaeJ rectifies translational problems that are not resolved by tmRNA and ArfA, rather than just being an alternative to tmRNA (2).

YaeJ homologs are also found in most eukaryotes, from yeast to humans. Release factor assays using *E. coli* S30 fractions rich in ribosomes have shown that ICT1, the human homolog of YaeJ, has codon-independent peptidyl-tRNA hydrolysis (PTH) activity via the GGQ motif, although there are no direct comparisons of ICT1 and YaeJ activity (15). Depletion of ICT1 using siRNA results in a reduction of mitochondrial protein synthesis, leading to a loss of cell viability as well as mitochondrial dysfunction (15,16). These findings indicate that ICT1 also functions as a stalled ribosome rescue factor in mitochondria.

The solution structure of ICT1 from *Mus musculus* was first determined by NMR (16). ICT1 can be divided into two parts: the structured GGQ domain containing a catalytic site at the N-terminus, and an unstructured basic residue-rich extension at the C-terminus. Thus, ICT1 completely lacks such structured domains at the C-terminus, present in RFs, that are required for stop codon recognition. The GGQ domain of ICT1 is virtually identical in its structural framework to that of RF, except for the region connecting β2 and β3, where RF has a six-residue π-HB turn, while ICT1 has a 10-residue α-helix (α_i_) (16,17). A structure-based sequence alignment suggested that the presence of α_i_ is conserved not only among ICT1 proteins from eukaryotes, but also among YaeJ proteins from bacteria (16).

Recently, a crystal structure of *E. coli* YaeJ, bound to the *T. thermophilus* 70S ribosome in complex with initiator 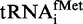 and a short mRNA, has given insight into the mechanism of YaeJ function (18). A section of the C-terminal extension of YaeJ (termed the C-terminal tail) forms a helical region, which lies in the mRNA entry channel, downstream of the A-site that is vacant in the 30S subunit. Accordingly, the C-terminal tail is thought to act as a sensor to discriminate between stalled and actively translating ribosomes. The fixed C-terminal tail is connected to the structured GGQ domain with a linker region that is poorly ordered. The GGQ domain of YaeJ is positioned in the A-site in a manner similar to that of RF, with the GGQ motif residues located at the peptidyltransferase center (PTC) of the 50S subunit.

**Figure 1.**
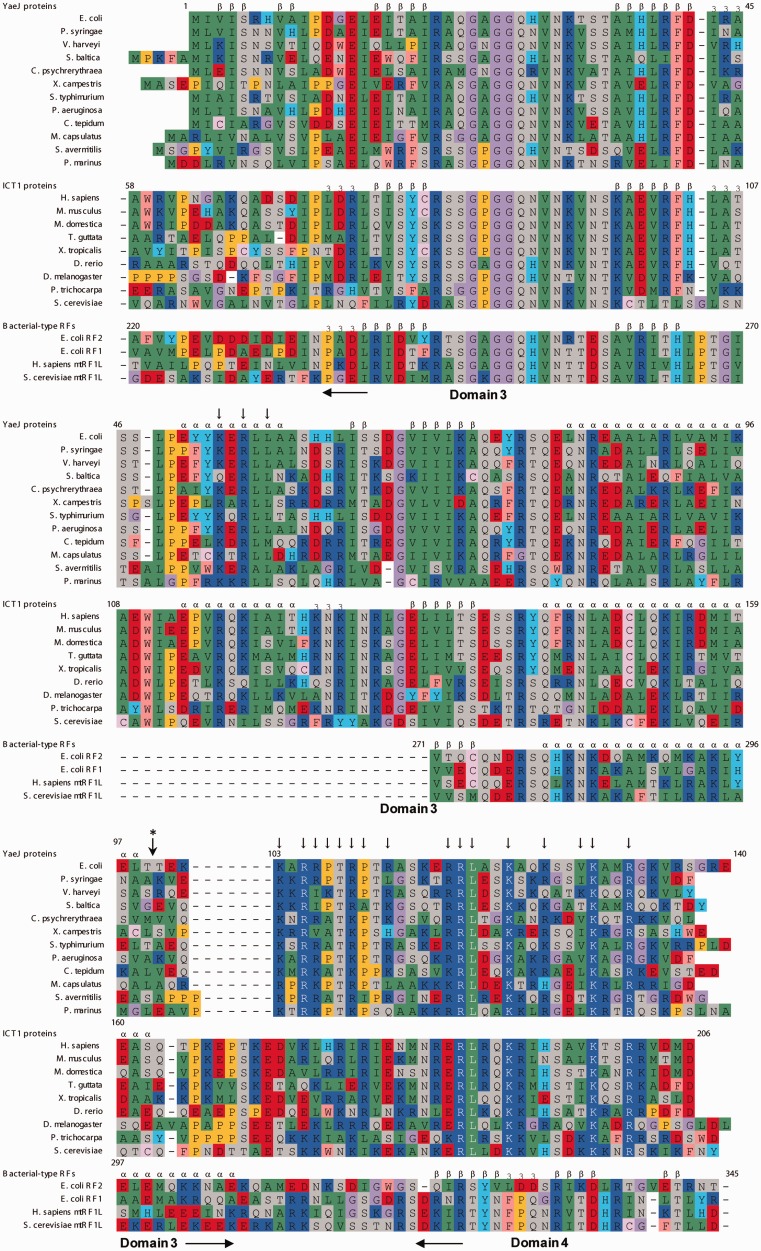
Sequence alignment of YaeJ proteins from bacteria, ICT1 proteins from eukaryotes and *E. coli*, and mitochondrial RFs. Secondary structure elements of the YaeJ structure determined in this study (PDB ID 2RTX), ICT1 (1J26) and the RF2 structure (1GQE) are indicated. The truncation position for the YaeJ protein used in the structural determination is marked by a vertical arrow with an asterisk. Vertical arrows indicate mutation positions, which are occupied by highly conserved residues (>90%) among all of the YaeJ proteins described in the article. Alignments are colored as follows: purple: glycine (G); yellow: proline (P); green: small and hydrophobic amino acids (A, V, L, I, M); pink: hydrophobic aromatic residues (F, W); gray: hydroxyl and amine amino acids (S, T, N, Q); red: negatively charged amino acids (D, E); blue: positively charged amino acids (K, R); pale pink: cysteine (C); cyan: histidine (H) and tyrosine (Y).

Although the co-crystal structure provides an important basis for understanding how YaeJ rescues stalled ribosomes, there is little biochemical data about interactions between YaeJ and stalled ribosomes in *E. coli*. Sequence alignment among YaeJ proteins from various Gram-negative bacteria shows the presence of a number of highly conserved residues that do not occur in RFs (Figure 1), which are candidates as being residues required for YaeJ-specific functions. However, the co-crystal structure can only explain the importance of some of these residues, as described below.

To identify residues required for YaeJ function in this study, we performed mutational studies of YaeJ to identify PTH activity towards non-stop mRNA using an *E. coli*-based reconstituted cell-free protein synthesis system. We also examined ribosome binding properties of YaeJ mutants using sucrose density gradient sedimentation. Furthermore, using heteronuclear nuclear magnetic resonance (NMR) methods, we determined the solution structure of the GGQ domain of YaeJ from *E. coli* to obtain detailed structural information for the mutational analysis, and compared it with that of human homolog ICT1. We found that ICT1 had similar PTH activity to that of YaeJ for stalled ribosomes from *E. coli*, whereas there were various differences in sequence and structure between ICT1 and YaeJ, except for the conserved residues that form a PTH catalytic site, including the GGQ motif residues (Figure 1). These findings indicate that residues involved in the sequence and structural differences between YaeJ and ICT1 are not important for YaeJ-specific PTH activity, consequently reducing the number of mutation targets to a large extent. This study revealed that only several highly conserved residues are required for YaeJ-specific PTH activity and ribosome binding of YaeJ. Notably, no apparent interaction sites for some of the important residues were found in the co-crystal structure. Biochemical findings from this study may assist in understanding how YaeJ enters the A-site of stalled ribosomes.

## MATERIALS AND METHODS

### Sequence alignment

Amino acid sequence alignments were performed using the ClustalW program (19) and then manually modified on the basis of the structures of YaeJ, ICT1 and RF, and on the results of the mutational study. Accession codes used in the sequence alignment were as follows: *E. coli* str. K-12 YaeJ, AAC73302.1; *Pseudomonas syringae* pv. tomato str. DC3000 YaeJ, AAO55337.1; *Vibrio harveyi* YaeJ, ABU73293.1; *Shewanella baltica* YaeJ, ACK48274.1; *Colwellia psychrerythraea* YaeJ, YP_271049.1; *Xanthomonas campestris* pv. *campestris* str. ATCC 33913 YaeJ, AAM39546.1; *Salmonella typhimurium* LT2 YaeJ, NP_459245.1; *Pseudomonas aeruginosa* PA01 YaeJ, NP_249559.1; *Chlorobium tepidum* TLS YaeJ, NP_662859.1; *Methylococcus capsulatus* str. Bath YaeJ, YP_112667.1; *Streptomyces avermitilis* MA-4680 YaeJ, NP_825123.1; *Prochlorococcus marinus* str. MIT 9313 YaeJ, NP_896036.1; *M.musculus* ICT1, NP_081005.1; *Homo sapiens* ICT1, NP_001536.1; *Monodelphis domestica* ICT1, XP_001377961.1; *Taeniopygia guttata* ICT1, XP_002190739.2; *Xenopus (Silurana) tropicalis* ICT1, XP_002940027.1; *Danio rerio* ICT1, XP_001922710.1; *Drosophila melanogaster* ICT1, NP_609416.1; *Populus trichocarpa* ICT1, XP_002329853.1; *Saccharomyces cerevisiae* ICT1, AAS56333.1; *E. coli* RF2, P07012; *E. coli* RF1, P0A7I1; *Homo sapiens* mtRF1L, NP_061914.3; *S. cerevisiae* mtRF1L, P30775.

### *Escherichia coli* strains

The *yaeJ*-deficient strain JW0187 (BW25113 *yaeJ*::FRT-Km^r^-FRT) derived from BW25113 was provided by the National BioResource Project (NBRP) (NIG, Japan) (20). The *yaeJ*-deficient (Δ*yaeJ*) strain used in this study was constructed by phage P1-mediated transduction used to introduce the *yaeJ* mutation from JW0187 into BL21 strains. The absence of the gene for YaeJ was confirmed by PCR and genome sequence analysis.

### Protein expression and purification of recombinant His-tagged proteins

The DNA sequences encoding the YaeJ protein and the truncated ICT1 protein lacking residues 1–29 (Δ29 ICT1) were amplified by PCR using the plasmid JW0187, from the ASKA library (21), and human *ICT1* cDNA (IRAK027I21), provided by the RIKEN BioResource Center through the NBRP (22), as templates, respectively. The primers used in this study are listed in Supplementary Table S1. The amplified DNA fragments were digested with NdeI (New England Biolabs) and XhoI (New England Biolabs) and then cloned into the expression vector pET26b (Novagen) as a fusion protein with a C-terminal His_6_-tag. The vectors harboring the *yaeJ* gene and the Δ29 ICT1 protein gene were transformed into *E. coli* BL21(DE3) pLysS cells (Novagen) and BL21-CodonPlus(DE3)-*RIL* cells (Novagen), respectively. Kanamycin and chloramphenicol resistant colonies were selected and grown in liquid Luria-Bertani (LB) medium. Protein expression was initiated at OD_600_ ∼ 0.6 by the addition of isopropyl-D-thiogalactopyranoside (IPTG; Wako) at a final concentration of 0.5 mM. Cells in which the recombinant protein was expressed for 5 h at 25°C were harvested by centrifugation (5000× *g*, 15 min, 4°C). The resulting pellet was resuspended in buffer A [50 mM HEPES–KOH (pH 7.6), 0.5 M KCl, 1 mM EDTA, 1 mM DTT, 1 mM aminoethylbenzylsulfonyl fluoride (AEBSF; Roche Applied Science)]. The recombinant protein was liberated by sonication and cell debris was removed by centrifugation (12 000× *g*, 30 min, 4°C). The supernatant was applied to a cOmplete His-Tag Purification Resin (Roche Applied Science). The recombinant proteins were eluted with buffer B [50 mM HEPES–KOH (pH 7.6), 0.5 M KCl, 1 mM EDTA, 1 mM DTT, 0.5 M imidazole]. Fractions containing the recombinant proteins were applied to a HiLoad 16/60 Superdex 200 prep grade column with buffer C (50 mM HEPES–KOH (pH 7.6), 0.3 M KCl, 1 mM DTT, 1% glycerol). After the eluted protein was concentrated, buffer was exchanged with buffer D (50 mM HEPES–KOH (pH 7.6), 0.1 M KCl, 10 mM MgCl_2_, 1 mM DTT) using an Amicon Ultra concentrator (Millipore). Protein concentration was determined using Quick start Bradford Dye Reagent (Bio-Rad) according to the manufacturer’s instructions using bovine serum albumin as standard.

### Site-directed mutagenesis of YaeJ and ICT1

Mutations were introduced into the *yaeJ* gene and human *ICT1* gene using overlap PCR or inverse PCR methods (23). The mutagenic oligonucleotide primers used in this study are listed in Supplementary Table S1. Amplification was performed using KOD Plus Neo polymerase (TOYOBO) and plasmids pET26b-*yaeJ* or pET-26b-Δ29 *ICT1* as the template. In the overlap PCR method, the amplicons were digested with DpnI (Roche Applied Science). In the inverse PCR method, the amplicons were treated with DpnI and T4 polynucleotide kinase (TAKARA) for 2 h at 37°C, and after purification, the PCR products were self-ligated for 30 min at 16°C using a DNA ligation kit (Ligation high, TOYOBO). Finally, the resulting samples were transformed into competent DH5α cells.

### *In vitro* translation using the PUREsystem

A PURExpress protein synthesis kit (New England Biolabs), based on PUREsystem technology, and the FluoroTect Green_Lys_ *in vitro* translation labeling system (Promega) were used for *in vitro* translation experiments. Template DNA fragments of YaeJ or the mutants were prepared using a two-step PCR reaction according to the manufacturer’s protocol, as described previously (13). Primer sequences are listed in Supplementary Table S1. The PCR products were purified using the Labo Pas PCR kit (Hokkaido System Science), and the resultant samples were treated with 4× sample buffer [125 mM Tris–HCl (pH6.8), 4% SDS, 10% glycerol, 10% 2-mercaptoethanol, 0.1% bromophenol Blue, 1× RNAsecure (Applied Biosystems)] and then analyzed using the NuPAGE Bis–Tris electrophoresis system (Invitrogen Life Technologies). Direct ‘in-gel’ detection of the proteins containing fluorescently labeled lysine residues was accomplished using ImageQuant LAS 4010 (GE Healthcare). The fluorescent signals were analyzed using the ImageQuant software (GE Healthcare). To determine the yield of YaeJ and its mutants, the proteins, along with the positive control protein dihydrofolate reductase (DHFR), were expressed and analyzed using the NuPAGE system. When the yield of DHFR was 200 μg/ml, the yields of YaeJ or its mutants were determined according to the relative band intensities quantified using ImageQuant LAS 4010, as described previously (13).

### PTH assay

YaeJ protein and its mutants were expressed using the PURExpress delta RF123 kit (New England Biolabs) as described above. Independently, using a non-stop template prepared from the *crp* gene, an *in vitro* translation reaction was performed for 15 min at 37°C to produce stalled ribosomes with peptidyl-tRNAs, to which the *in vitro* translation mixture containing 2.5 μM of YaeJ or the mutant was directly added. The resulting mixture was incubated for 5 min and then analyzed by NuPAGE as described above. Band intensities corresponding to CRP protein or CRP-tRNA were quantified using ImageQuant software, and the efficiency of PTH was calculated as the ratio of CRP protein to CRP-tRNA plus CRP protein. The ribosome concentration in the reaction solution of PURExpress was 2.5 μM.

### Sucrose density gradient centrifugation

The *E. coli* Δ*yaeJ* strain was grown in LB medium to an OD_600_ of 0.6∼0.8 (log phase). Harvested cells were washed once with Buffer I [20 mM Tris–HCl (pH7.5), 15 mM magnesium acetate, 100 mM ammonium acetate]. The pellet was then resuspended in Buffer II [20 mM Tris–HCl (pH 7.6), 15 mM magnesium acetate, 100 mM ammonium acetate, 1 mM AEBSF and 1 mM DTT], mixed with an equal volume of glass beads (250–425 microns, Fuji Rika Kogyo), and vortexed 15 times for 30 sec. After removal of cell debris by centrifugation at 12 000× *g* for 30 min at 4°C, the supernatant [20 *A*_260_ units/ml (0.80 mg/ml)] was mixed with the purified recombinant protein, of which the final concentration was 15 μM. The mixed supernatant was then layered onto a linear 5–20% sucrose density gradient made with association buffer [10 mM Tris–HCl (pH 7.5), 50 mM NH_4_Cl, and 10 mM MgCl_2_]. The mixed supernatant was layered on top of the sucrose gradients and centrifuged at 112 700× *g* in a P28S rotor (Hitachi Koki) for 3 h at 4°C. The absorbance of each fraction was measured at 260 nm using a GeneQuant pro spectrophotometer (GE Healthcare).

### Immunoblotting

The proteins (10 μg/lane) were separated by 15% sodium dodecyl sulfate-polyacrylamide gel electrophoresis (SDS-PAGE) and subsequently transferred onto polyvinylidene fluoride (PVDF; GE Healthcare) membrane. The membrane was then blocked with 3% skim milk in PBS containing 0.1% Tween 20 at room temperature for 30 min. The membrane was then incubated with a mouse polyclonal antibody against the His_6_-tag (MBL, 1:5000) overnight at 4°C, and with horseradish peroxidase-conjugated goat anti-mouse IgG antibody (Cell Signaling Technology, 1:4000) for 1 h at room temperature. Can Get Signal Immunoreaction Enhancer Solution (TOYOBO) was used to dilute the antibodies according to the manufacturer’s instructions. The proteins were visualized using an enhanced chemiluminescence kit according to the manufacturer’s instructions (Western Lightning ECL Pro, PerkinElmer), and band intensities corresponding to YaeJ or its mutants were quantified using ImageQuant software.

## RESULTS

### Comparison of solution structures of the GGQ domains of YaeJ and ICT1

We determined the solution structure of the GGQ domain of YaeJ from *E. coli* using conventional heteronuclear NMR methods (Supplementary data). The protein encoded by the *E. coli yaeJ* gene is composed of 140 residues. Based on the NMR structure of ICT1, we designed a 109-residue truncated protein, which lacks the C-terminal region from residue 110 to the last residue (Figure 1). ^15^N- and ^13^C/^15^N-labeled protein samples for NMR measurements were prepared using a cell-free protein expression system (24,25). The expressed protein had an artificial tag-derived sequence (12 residues) at the C-terminus, which was derived from the expression vector, and was consequently composed of 121 residues. Tertiary structures were calculated using the CYANA software package (26,27) based on a total of 2592 NOE-derived distance restraints and 82 backbone torsion angle restraints. Statistics for the structure determination are summarized in Supplementary Table S2.

**Figure 2.**
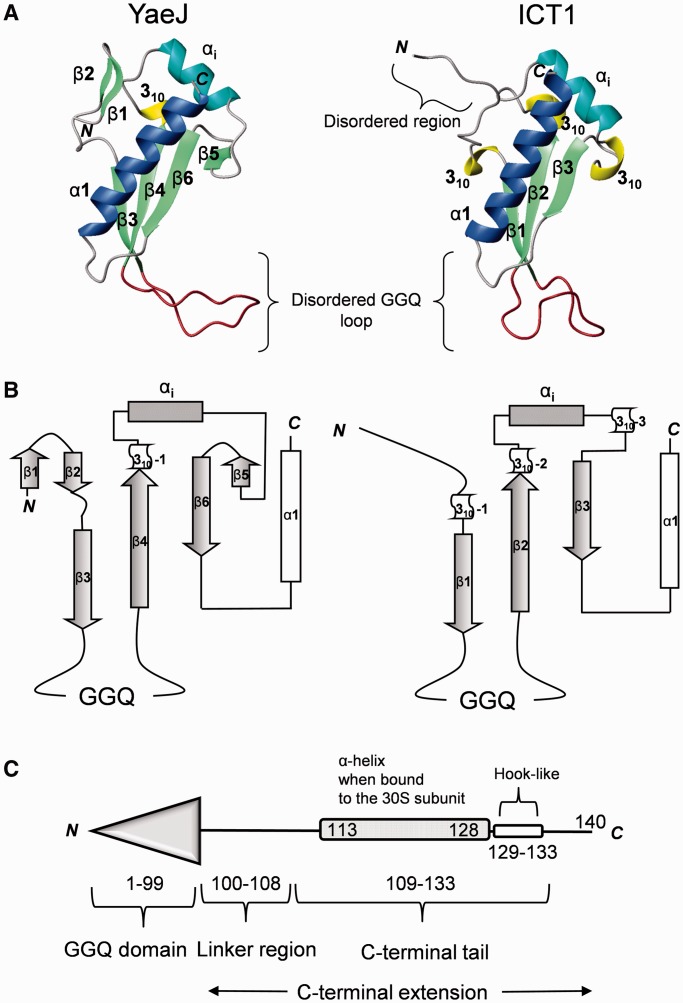
Comparison of the GGQ domains of YaeJ and ICT1. (**A**) Ribbon diagrams of the GGQ domain structures of *E. coli* YaeJ (PDB ID 2RTX; residues 1–109) and mouse ICT1 (PDB ID 1J26; residues 63–162). The α-helices α1 and α_i_ are shown in blue and cyan, respectively. The 3_10_ helices, β-strands, and the GGQ loop are shown in yellow, light green and brown, respectively. Note that the GGQ loops are disordered in both structures and thus their shapes vary. (**B**) Topology of their GGQ domain structures. (**C**) Schematic representations of YaeJ according to the co-crystal structure (PDB ID 4DH9).

The NMR results showed that the structure of the GGQ domain consists of a four-stranded antiparallel β-sheet side-by-side with a small antiparallel β-sheet and an α-helix, with the topology β1-β2-β3-β4-3_10_-α_i_-β5-β6-α1 (Figure 2 and Supplementary Figure S1). The first small β-sheet consists of two antiparallel β-strands (β1: 3–5, β2: 8–9), while the second β-sheet consists of four antiparallel β-strands (β3: 16–20, β4: 36–42, β5: 64–65, β6: 69–74). The strands β3 and β4 are connected by a disordered loop (21–35) including the ^26^GGQ^28^ residues, which is termed the GGQ loop. The strands β4 and β6 are connected by an inserted region containing a 3_10_ helix (43–45), an α-helix (α_i_: 50–58) and β5, as described in detail below. Following β6, a long α-helix (α1: 82–98) lies on the second β-sheet. As a result, the structured region of the YaeJ protein, corresponding to the GGQ domain, ranges from Met1 to Thr99. This structural framework is virtually identical to that of a YaeJ homolog from *P. syringae* (28), but relative positions of the small β-sheet and β6 differ between the two proteins (Supplementary Figure S2A).

A structural comparison between YaeJ and its eukaryotic ortholog ICT1 from a mouse showed that the catalytic GGQ domain of YaeJ is essentially the same as that of ICT1, whereas structural differences are found in the N-terminal and the inserted regions (Figure 2A and B). At the N-terminus, YaeJ has a small β-sheet within the first nine residues, whereas ICT1 has an ordered stretch following a disordered region. Both proteins have an inserted region containing α_i_ between the β-strands (β4 and β6 in YaeJ; β2 and β3 in ICT1), which is the region most different in structure from that of the GGQ domain structure of RF. However, at the end of α_i_, a β-strand occurs in YaeJ, whereas ICT1 has a 3_10_ helix. Sequence alignment suggested that these YaeJ- and ICT1-specific features of the GGQ domains are preserved in bacteria and eukaryotes, respectively (Figure 1).

The crystal structure of full-length YaeJ bound to the *T. thermophilus* 70S ribosome showed a bound form of YaeJ, of which the GGQ domain is positioned in the A-site in a manner similar to the GGQ domain of RF (18). Comparison of the free and bound forms of the GGQ domain of YaeJ revealed that the largest difference in structure occurs in the GGQ loop: it is unstructured in the free form, whereas in the bound form, a section including the GGQ motif residues folds into an α-helix to form a catalytic site, as observed in free and bound forms of RFs (29,30). Except for the GGQ loop, the two structures of the GGQ domain appear to be basically similar (Supplementary Figure 2B). However, there is a structural difference in β4, where in the bound form, the side chains of charged residues are inside the structure and those of hydrophobic residues are outside.

### Human ortholog ICT1 is an efficient ribosome rescue factor for stalled ribosomes from *E. coli*

**Figure 3.**
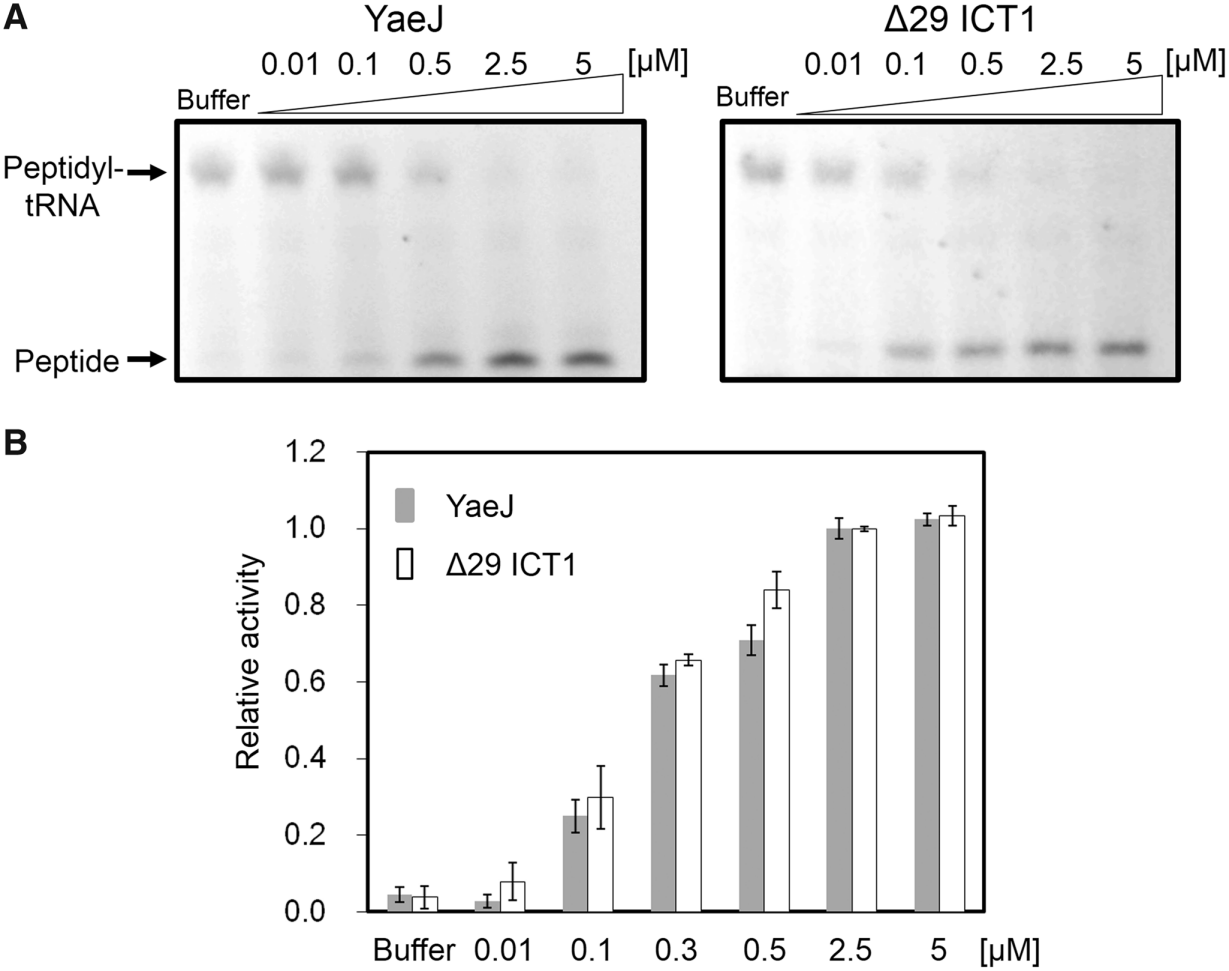
YaeJ and ICT1 have similar PTH activity towards stalled ribosomes from *E. coli*. (**A**) *In vitro* translation of the non-stop template (non-stop) with recombinant His-tagged YaeJ (left) or ICT1 protein (Δ29 ICT1) (right). Each recombinant protein was added to the solution in which a 15-min *in vitro* translation reaction had been performed using the non-stop templates. The final concentration of YaeJ or Δ29 ICT1 is shown in each lane. (**B**) Extent of PTH given as the ratio of the band intensity of released peptides to that of peptidyl-tRNA plus that of released peptides at various protein concentrations. Released peptides indicate CRP proteins and peptidyl-tRNA indicates CRP-tRNA, respectively. Data are presented as mean ± SD for three independent experiments.

It has been reported that ICT1 from humans has PTH activity that hydrolyzes peptidyl-tRNA at the P-site of ribosomes stalled on a nonstop mRNA using *E. coli* S30 fractions, but it is unclear whether ICT1 has similar PTH activity to that of YaeJ (15). We compared PTH activities of YaeJ and ICT1 towards stalled ribosomes using a cell-free protein synthesis system reconstituted from *E. coli* purified components (PUREsystem). Using a non-stop template that contains the *crp* gene lacking a stop codon (31), an *in vitro* translation reaction was performed for 15 min to produce stalled ribosomes with peptidyl-tRNAs (CRP-tRNAs). Various concentrations of recombinant YaeJ or ICT1 (with a His-tag at the C-terminus) were added to these ribosomes. The recombinant ICT1 protein (Δ29 ICT1) was designed as a truncated protein lacking the N-terminal 29 residues, which would function as a signal for import into mitochondria. The *in vitro* translation products were analyzed using the NuPAGE Bis–Tris electrophoresis system, because one of the products, peptidyl-tRNA, is stable in this neutral gel (31,32). Products labeled fluorescently with tRNA^Lys^ charged with a fluorescently labeled lysine were detected and quantified using ImageQuant LAS 4010 (Figure 3A). The ratio of the band intensity of released peptides to that of peptidyl-tRNA plus that of released peptides was defined as relative PTH activity for stalled ribosomes. Thus, 100% of the relative activity indicated that peptidyl-tRNAs on stalled ribosomes were fully hydrolyzed. Figure 3B shows a comparison of the PTH activity of YaeJ and ICT1 at various concentrations. These results showed that ICT1, which functions in mitoribosomes, can rescue stalled ribosomes from *E. coli* in a manner as efficient as YaeJ.

These results also indicated that none of the residues involved in the sequence and structural differences between ICT1 and YaeJ contribute significantly to YaeJ-specific PTH activity. Particularly, the YaeJ-specific features of the GGQ domains, namely the N-terminal region and the end of α_i_, are not important for PTH activity. Residues required for YaeJ-specific PTH activity were expected to occur at the corresponding positions in ICT1. Thus, the target residues for mutation we focused on were those that were highly conserved in bacteria and had side chains on the outside, that differed from those in RF, and that were found at the corresponding position in ICT1 in the range of the GGQ domain.

### Mutations in the GGQ domain

In this mutational study, YaeJ mutants were expressed on a small scale with no tag sequence using the PURE system. The concentration of each YaeJ variant was adjusted to 2.5 µM, at which wild-type YaeJ can fully hydrolyze peptidyl-tRNAs on stalled ribosomes under the experimental conditions examined (Figure 3B).

**Figure 4.**
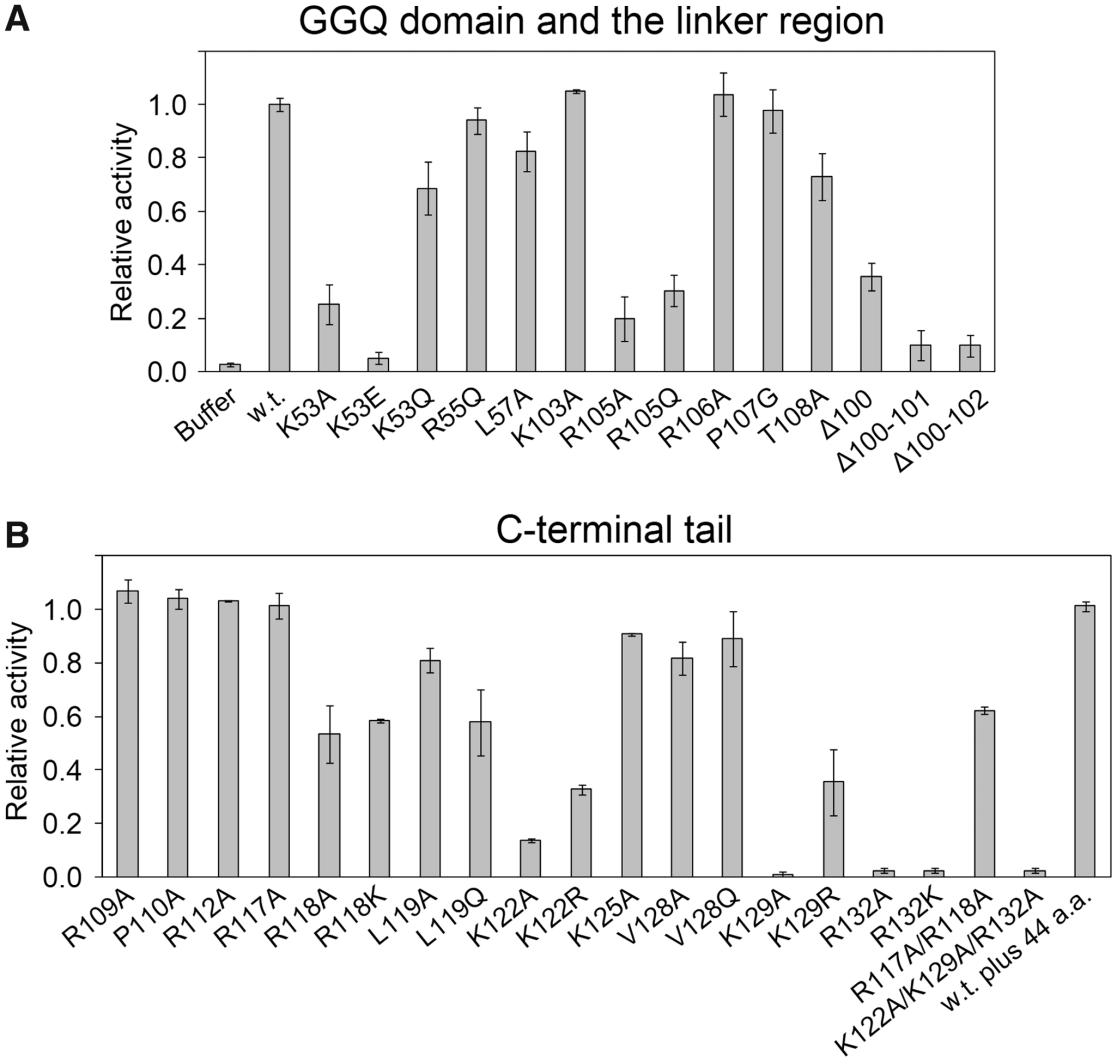
Effect of mutations of YaeJ on PTH activity. Wild-type YaeJ and the mutants in which well-conserved residues were substituted were expressed using the *in vitro* translation system. The *in vitro* translation reaction mixture containing YaeJ or a YaeJ mutant was directly added to another solution in which a preliminarily 15-min *in vitro* translation reaction had been performed using the non-stop template. The final concentration of YaeJ and the mutants was adjusted to 2.5 μM. Data are presented as mean ± SD for three independent experiments. (**A**) Mutations in the GGQ domain and the linker region. (**B**) Mutations in the C-terminal tail. The term ‘w.t. plus 44 a.a.’ means a mutant in which 44 residues derived from the expression plasmid pET15b were added to the C-terminus of the wild-type, as described previously (12).

In the GGQ domain, a detailed comparison of the sequence and structure of YaeJ and ICT1 limited the number of candidate residues for mutation. Candidate residues were Lys53, Arg55 and Leu57, located in the inserted α-helix, α_i_, which is the most characteristic feature of YaeJ and ICT1 but does not appear in RF. In YaeJ proteins, position 53 is usually occupied by Lys (or occasionally Arg or Gln), and position 57 is occupied by Leu (or occasionally Met). Position 55 is always occupied by Arg (Figure 1). Substitution of Lys53 with Ala or Glu resulted in significant decreases in PTH activity (25% and 5% of the wild-type PTH activity, respectively), whereas substitution with Gln showed 70% PTH activity (Figure 4A). Substitutions of Arg55 and Leu57 with Gln and Ala, respectively, only had a slight effect on PTH activity.

To examine whether the significant decrease in PTH activity by the mutations at position 53 was caused by significant structural changes, we attempted to measure circular dichroic spectra for comparison between the mutants and the wild type. However, the corresponding overexpressed recombinant proteins became easily insoluble, and thus could only be obtained in very small quantities (data not shown). Instead, we compared the surface electrostatic potential around positions 53 and 57 in the GGQ domain structures of YaeJ and ICT1. The distributions differed substantially between YaeJ and ICT1 (Supplementary Figure S3A). Thus, considering that YaeJ and ICT1 have similar PTH activity, it appears that Lys53 or the inserted α-helix does not directly contribute to PTH activity. These findings, along with comparisons in sequence and structure between YaeJ and ICT1, showed that the GGQ domain has no YaeJ-specific residues that are important for PTH activity.

In light of the YaeJ structure in the free form, Lys53 and Arg55 are expected to play a role in structural stability (Supplementary Figure S3B). The hydrophobic region of the side chain of Lys53 interacts with the side chains of Ile43 and Leu48 buried in the structure, while the ε-amino group of Lys53 appears to form a hydrogen bond with the O of Ser46 and/or Ile43. In α_i_, the side chain of Arg55 contacts the aromatic ring of Tyr51, and this position is highly conserved as Tyr or Phe (Figure 1).

### Mutations in the C-terminal tail

According to the co-crystal structure, the C-terminal extension, which is flexible in the free form, can be divided into two regions (Figure 2C). One is termed the C-terminal tail, which forms an α-helix that is positioned in the mRNA entry channel. The other is termed the linker region, connecting the GGQ domain and the C-terminal tail, and appears to be unstructured even in the bound form, where it exhibits poor electron density.

In the C-terminal tail, each of the well-conserved residues, Arg109, Pro110, Arg112, Arg117, Arg118, Leu119, Lys122, Lys125, Val128, Lys129 and Arg132, was substituted with Ala or other amino acids (Figure 1). Decreases in PTH activity were observed when mutations were made at Arg118, Leu119, Lys122, Lys129 and Arg132 (Figure 4B). The order of the degree of decrease was Arg132 and Lys129 (∼0%) < Lys122 (∼25%) < Leu119 and Arg118 (∼60%). The circular dichroic spectra of the corresponding recombinant mutants were similar to those of the wild type, showing that the mutations do not have significant effects on the structural GGQ domain (data not shown). As for Lys122 and Lys129, substitution with the other basic residue, Arg, decreased the PTH activity less significantly than Ala, suggesting that the electric charge of the side chain plays a role in the interaction with some factor of the 30S subunit. In contrast, substitution of Arg132 with Lys decreased the PTH activity as severely as Ala, suggesting that the side chain of Arg132 itself may be involved in such interactions. A previous study showed that a mutant in which the last 10 residues were truncated had no detectable PTH activity (13), which can be explained by loss of Arg132 in the mutant.

No substantial decrease in PTH activity was observed when mutations were introduced at Arg109, Pro110, Arg112, Lys117, Lys125 or Val128 (Figure 4B). These results showed that in the C-terminal tail, the five residues Arg118, Leu119, Lys122, Lys129 and Arg132 are required for PTH activity of YaeJ, all of which are completely conserved among YaeJ proteins from various bacteria (Figure 1).

### Mutations in the linker region

In the linker region connecting the GGQ domain and the C-terminal tail, each of the residues Lys103, Arg105, Arg106, Pro107 and Thr108 were changed to Ala or other amino acids. Substitution of Arg105 with Ala or Gln resulted in a significant decrease in PTH activity, showing that Arg105 is involved in PTH activity (Figure 4A). Substitution of Thr108 with Ala resulted in a moderate decrease in PTH activity. No substantial decrease in PTH activity was observed when a mutation was made at Lys103, Arg106 or Pro107 (Figure 4A).

### Deletion mutations in the C-terminal region

The co-crystal structure of YaeJ and the 70S ribosome suggested that a certain minimum length of linker between the GGQ domain and the C-terminal tail is necessary for YaeJ function on the stalled ribosome. To confirm the importance of the length of the linker region, we generated deletion mutants. A deletion mutant lacking Thr100 still showed 35% PTH activity, while a deletion of Thr100 and Glu101 (Δ100–101 mutant) severely decreased the PTH activity (Figure 4A). These results showed that a certain length between the GGQ domain and the C-terminal α-helix is required for PTH activity, although a single-residue deletion is tolerated to some extent.

### Ribosome binding

**Figure 5.**
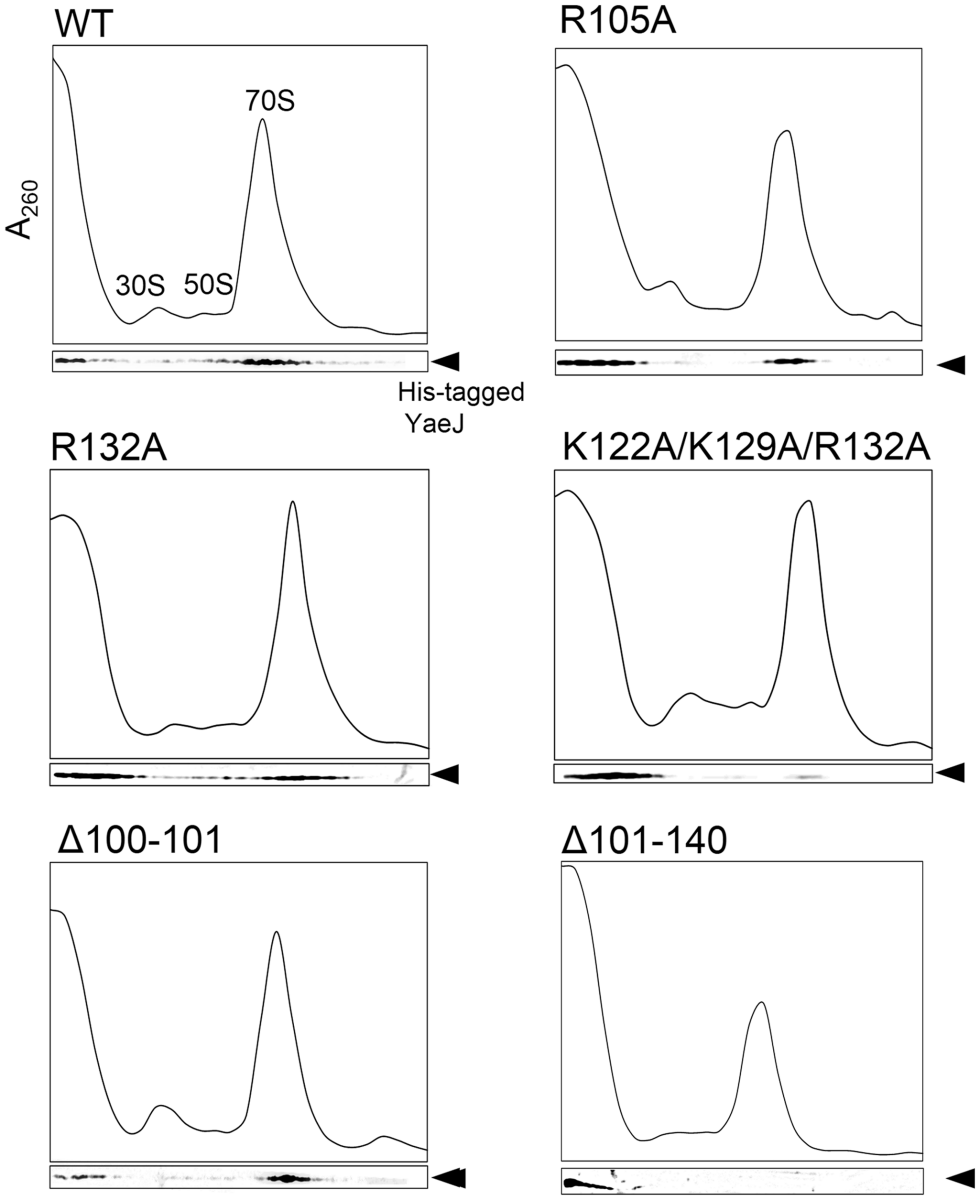
Ribosome-binding properties of His-tagged recombinant YaeJ protein and the mutants. After separation through 5–20% sucrose gradients, fractions were analyzed by western blotting using an anti-His antibody. Results are representative of three independent experiments.

To examine the effect of these mutations on YaeJ binding to ribosomes, we performed sucrose density gradient centrifugation experiments using the YaeJ-depleted S30 fraction (33). Wild-type YaeJ or its mutants, each having a His-tag at the C-terminus, were added to cell lysate from the *yaeJ*-deficient (Δ*yaeJ*) strain, and the mixture was separated by sucrose density gradient centrifugation. The distribution of wild-type YaeJ or each mutant was examined by western blotting with His-tag antibodies, and the signal intensity was quantified using a digital imaging system. For the wild type, the signal intensity ratios of soluble fractions, 30S + 50S fractions, and 70S fractions to the total signal intensity were 26%, 6% and 64%, respectively (Figure 5 and Table 1). For the K122A mutant, the corresponding ratios were 65%, 8% and 26%, respectively. These results suggested that this mutation in the C-terminal tail decreased the binding efficiency of YaeJ to ribosomes. A similar result was obtained for the R132A mutant. A triple mutant of K122A, K129A and R132A, which showed no detectable PTH activity, had even less binding efficiency to 70S ribosomes than the K122A or R132A single mutants. The R105A mutant also had a decreased binding efficiency to 70S ribosomes.

**Table 1.** Ribosome-binding properties of YaeJ mutants

Mutants	Fractions (%)			
	Soluble	30S + 50S	70S	Polysome
WT	26 ± 3	6 ± 3	64 ± 2	4 ± 2
R105A	71 ± 15	4 ± 3	24 ± 14	1 ± 2
K122A	65 ± 10	8 ± 2	26 ± 11	1 ± 1
R132A	61 ± 7	6 ± 3	32 ± 6	1 ± 1
Triple	75 ± 16	7 ± 4	17 ± 13	1 ± 2
Δ100–101	18 ± 6	8 ± 7	69 ± 13	5 ± 7
Δ101–140	100 ± 2	0	0	0

Triple: K122A/K129A/R132A.

Fractions (%) indicate the ratio of the signal intensity of each fraction to the total signal intensity. The signals of each mutant are shown in Figure 5. Data are presented as mean ± SD for three independent experiments.

To verify whether the GGQ domain alone can bind to ribosomes, we made a His-tagged Δ101–140 mutant that completely lacked the C-terminal extension from residues 101–140. This mutant showed no detectable PTH activity, and signals were detected only in the soluble fractions (Figure 5 and Table 1), suggesting that the GGQ domain alone has no capacity to bind to ribosomes, and the association of YaeJ with ribosomes depends mainly on the C-terminal extension.

For the Δ100–101 mutant, the linker region was shortened by two residues. This mutant showed no detectable PTH activity, whereas it had a ribosome binding efficiency similar to that of the wild type. The signal intensity ratios of soluble fractions, 30S + 50S fractions and 70S fractions to the total signal intensity were 18%, 8% and 69%, respectively (Figure 5 and Table 1). Presumably, the C-terminal tail of this deletion mutant manages to be placed in the correct position in the 30S subunit, whereas the GGQ domain cannot reach the PTC of the 50S subunit because of the insufficient length of the linker region.

### Sequence alignment of the C-terminal extensions of YaeJ and ICT1 and confirmation of the alignment by mutational analysis of ICT1

In the C-terminal extension, an automatic sequence alignment between YaeJ proteins from bacteria and ICT1 proteins from eukaryotes could not be achieved in the CLUSTAL program, as shown previously (16), because the C-terminal region differs in length between these proteins and has an abundance of basic residues. However, clarification of the residues required for PTH activity of YaeJ allowed for a meaningful sequence alignment in the C-terminal extension between the YaeJ and ICT1 proteins, consequently revealing sequence similarity in the last 25 residues of the C-terminal extension (Figure 1).

**Figure 6.**
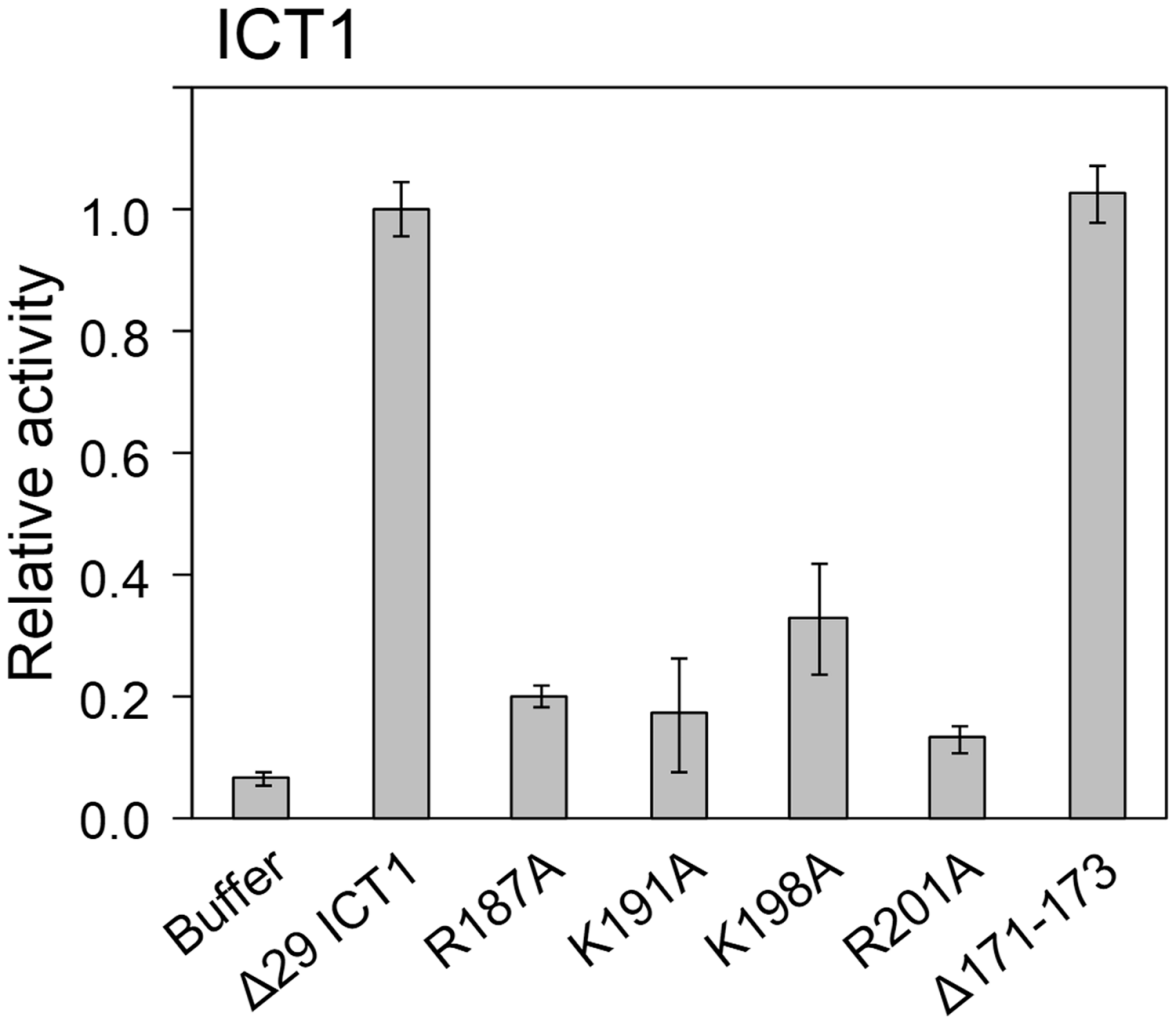
*In vitro* translation of the non-stop template (non-stop) with recombinant His-tagged ICT1 protein (Δ29 ICT1) and its mutants. The recombinant protein was added to the solution in which a 15-min *in vitro* translation reaction had been performed using the non-stop templates. Data are presented as mean ± SD from at least three independent experiments.

To confirm whether the sequence alignment of the C-terminal extension was appropriate, we introduced mutations into the C-terminal extension of human ICT1. Arg187, Lys191, Lys198 and Arg201 in ICT1 correspond to Arg118, Lys122, Lys129 and Arg132 in the C-terminal tail of YaeJ, respectively (Figure 1), all of which were found to be required for the PTH activity of YaeJ. These residues are conserved among not only YaeJ proteins, but also ICT1 proteins. Substitution of each of the four residues with Ala in ICT1 resulted in significant decreases in PTH activity (Figure 6). The sequence alignment showed that the linker region of ICT1 is six residues longer than that of YaeJ. Thus, since YaeJ and ICT1 have a similar level of PTH activity, a longer linker region is likely to be tolerated without loss of PTH activity. To confirm the redundancy of the length of the linker region, we shortened the linker region of ICT1 by three residues, Glu171, Asp172 and Val173. This mutant had a similar level of PTH activity to the wild type. Taken together, these results indicate that the sequence alignment is appropriate and that YaeJ function requires a minimum length of the linker region.

## DISCUSSION

**Figure 7.**
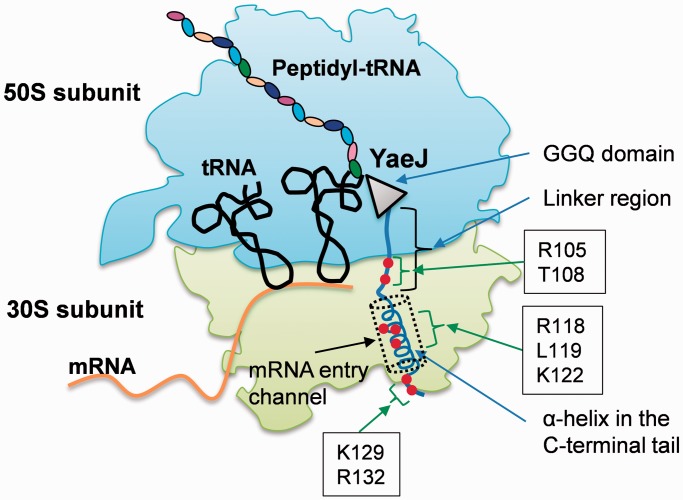
Mapping of residues required for PTH activity in YaeJ, as indicated by this study, onto a schematic representation of the YaeJ structure on a stalled ribosome. The schematic is based on the co-crystal structure of *E. coli* YaeJ and *T. thermophilus* 70S ribosome in complex with non-stop mRNA (PDB ID 4DH9). This study also showed that there is a minimum length for the linker region to achieve PTH activity. Note that the coordinates of residues 134–140 in the C-terminal extension are missing in the co-crystal structure.

This mutational study revealed residues required for YaeJ function on stalled ribosomes. The GGQ domains of YaeJ and ICT1 have essentially the same residues as RF forming a catalytic site of PTH activity. These residues are concentrated in the GGQ loop and the α1/β6 loop (Figure 1) (16). However, the GGQ domain of YaeJ has no YaeJ-specific residues that are important for PTH activity, conversely indicating that the mechanism by which YaeJ functions on stalled ribosomes depends predominantly on the C-terminal extension. In the C-terminal extension, Arg118, Leu119, Lys122, Lys129 and Arg132 in the C-terminal tail, and Arg105 (and Thr108 to a lesser extent) in the linker region are involved in PTH activity, all of which are completely conserved among bacteria (Figure 7). There is also a minimum length for the linker region connecting the GGQ domain and the C-terminal tail to achieve PTH activity. The importance of the C-terminal tail as well as the length of the linker region is basically consistent with the findings from the co-crystal structure analysis of YaeJ bound to 70S ribosomes (18). Mutations of these residues resulted in decrease in ribosome-binding efficiency, which are linked to decreases in PTH activity. These findings suggest that the mutants may have greater difficulty entering the A-site on stalled ribosomes than the wild type, or even if the mutants are able to enter the A-site, they are more easily released from the ribosomes or less efficient at forming a catalytic center for PTH activity.

According to the co-crystal structure, decreases in PTH activity or ribosome-binding efficiency by mutations at three (Arg118, Leu119 and Lys122) of five relevant residues in the C-terminal tail could be explained by a loss of specific interactions with ribosomal RNA and/or proteins inside the mRNA entry channel in the 30S subunits. Arg118 makes a π-cation interaction with Gua530 in 16S RNA, inducing the switch from ‘syn’ to ‘anti’ conformation, as is seen in the tRNA binding to the decoding region (18). Leu119 and Lys122 have the potential to interact with Cyt1397 and Uri1196, respectively. In contrast, although Pro110 and Arg117 have specific interactions with the ribosome in the co-crystal structure, an amino acid substitution at each residue had no significant effects on PTH activity. The side chain of Arg117 interacts with Cyt518 and Ser50 of ribosomal protein S12, while Ser50 contacts N6 of Ade1492 upon decoding. Pro110 stacks with Ade1493, acting as a hinge to fix the conformation of the tail. The positions of Ade1492 and Ade1493 in the complex are different from those induced by tRNA binding. The discrepancies between the co-crystal structure and the present biochemical study might be attributed to the fact that the co-crystal is made by components from heterologous sources, such as *E. coli* YaeJ and *T. thermophilus* ribosome.

With regard to the other mutations, the co-crystal structure provides no adequate explanations for the decreased ribosome binding efficiency or decreased PTH activity. Lys129 and Arg132, which follow the α-helix region of the C-terminal tail, entirely pass through the channel. Neither residue has any apparent interaction with rRNA or a ribosomal protein, although the 16S rRNA central pseudoknot lies in the vicinity of these residues. Coordinates for the residues 134–140 are missing in the co-crystal structure. Arg105 and Thr108 in the linker region have no obvious interaction with the ribosome. Thus, Lys129, Arg132 and Arg105 might play some role when YaeJ enters the A-site on stalled ribosomes. This process would include an accommodation-like step of YaeJ into the A-site, as seen in *trans*-translation mediated by tmRNA/SmpB. The C-terminal tail of SmpB is similar to that of YaeJ, in that it is rich in basic residues and flexible in solution while it occupies the decoding center leading to the mRNA entry channel in the stalled ribosome (34). Several lines of evidence have shown that the C-terminal tail of SmpB plays an essential role in accommodating alanyl-tmRNA/SmpB into the A-site in the absence of mRNA (35–37). In the case of YaeJ, some portion of its C-terminal extension might play a key role in an accommodation-like step. However, the possibility cannot be excluded that decreases in ribosome binding by these mutations are due to unexpected interactions with ribosomes or some other factors.

These findings raise the question of how the C-terminal tail enters the mRNA entry channel. The fact that a YaeJ mutant, in which as many as 44 residues were added to the C-terminus, had a similar level of PTH activity to that of the wild type (Figure 4) raises the possibility that the C-terminal tail goes through a gap into the interior of the mRNA entry channel. The gap might be wider or looser in stalled ribosomes than in translating ribosomes, so that the C-terminal tail could easily bind to the mRNA entry channel, forming an α-helix. Further studies will be required to elucidate the molecular mechanism by which YaeJ enters the A-site of stalled ribosomes, and specifically which steps YaeJ executes on stalled ribosomes. This would reveal the unique role of YaeJ and how it differs from that of tmRNA/SmpB. This study also established the sequence alignment between bacterial YaeJ and eukaryotic ICT1 proteins. All of the residues in the C-terminal tail required for YaeJ-specific PTH activity are also conserved among ICT1 proteins. It would be interesting to examine whether ICT1 functions on stalled mitochondrial ribosomes in the same manner as YaeJ, despite the various differences in the translation apparatus between bacteria and mitochondria.

## ACCESSION NUMBERS

The sequence-specific resonance assignments of the *E. coli* YaeJ protein have been deposited in the BioMagResBank database (accession number 11534). The atomic coordinates of the 20 energy-minimized CYANA conformers of the YaeJ protein have been deposited in the RCSB Protein Data Bank (PDB) under PDB code 2RTX.

## SUPPLEMENTARY DATA

Supplementary Data are available at NAR Online, including [38–51].

Supplementary Data
