# Supplementary material for: Identification of residues required for stalled-ribosome rescue in the codon-independent release factor YaeJ
Source: Nucleic Acids Res. 2013 Dec 9;42(5):3152–63. doi: 10.1093/nar/gkt1280 (PMC3950681; doi:10.1093/nar/gkt1280)
Supplement: Supplementary Data [file supp_42_5_3152__index.html]

Identification of residues required for stalled-ribosome rescue in the codon-independent release factor YaeJ — Identification of residues required for stalled-ribosome rescue in the codon-independent release factor YaeJ — Supplementary Data 

# Identification of residues required for stalled-ribosome rescue in the codon-independent release factor YaeJ

## Supplementary Data

files

**Files in this Data Supplement:**

- Supplementary Data - pdf file
