# Supplementary material for: Identification of residues required for stalled-ribosome rescue in the codon-independent release factor YaeJ
Source: Nucleic Acids Res. 2013 Dec 9;42(5):3152–63. doi: 10.1093/nar/gkt1280 (PMC3950681; doi:10.1093/nar/gkt1280)
Supplement: Supplementary Data [file supp_gkt1280_nar-03005-r-2013-File009.pdf]

SUPPLEMENTARY DATA

Solution structure of the GGQ domain of the YaeJ protein from *E. coli*

MATERIAL AND METHODS

Protein expression and purification

The DNA encoding the region of the YaeJ protein ranging from Met1 to Arg109 was subcloned by PCR. This DNA fragment was cloned into the expression vector pCR2.1 (Invitrogen) as a fusion with a C-terminal His_6-tag and a TEV protease cleavage site. The $^{13}C/^{15}N$-labeled fusion protein was synthesized by the cell free protein expression system as described previously (24,25,38). The protein purification has been described previously (16). For NMR measurements, the purified protein was concentrated to ~1.0 mM in $^{1}H_2O/^{2}H_2O$ (9:1), 20 mM Tris-d_{11}-HCl buffer (pH 7.0), 100 mM NaCl, 1 mM 1,4-DL-dithiothreitol-d_{10} (*d*-DTT), and 0.02% NaN_3.

NMR spectroscopy, structure determination, and structure analysis

All NMR measurements were performed at 23°C on a Bruker AVANCE 700 spectrometer equipped with a triple-resonance CryoProbe. Sequence-specific backbone chemical shift assignments (39) were made with the $^{13}C/^{15}N$-labeled sample, using standard triple-resonance experiments (40,41). Assignments of side chains were obtained from HBHA(CO)NH, HC(CO)NH, C(CO)NH, HC(C)H-TOCSY, and HC(C)H-COSY spectra. ^{15}N- and ^{13}C-edited NOESY spectra with an 80 ms mixing time were used to obtain distance restraints. The spectra were processed with the program NMRpipe (42).

The program KUJIRA, which was created on the basis of NMRView (43), was used for spectral visualization and analysis (44).

Automated NOE cross-peak assignments (27,45) and structure calculations with torsion angle dynamics (26) were performed using the software package CYANA 3.93 (46). Peak lists of the two NOESY spectra were generated as input with the program NMRView. The input further contained the chemical shift list corresponding to the sequence-specific assignments. Dihedral angle restraints were derived using the program TALOS (47). No hydrogen bond restraints were used.

A total of 100 conformers were calculated independently. The 20 conformers with the lowest final CYANA target function values were energy-minimized in a water shell with the program OPALp (48) using the AMBER force field (49). The structures were validated using PROCHECK-NMR (50). The program MOLMOL was used to analyze the resulting 20 conformers and to prepare drawings of the structures (51).

RESULTS

Resonance assignments and structure determination

Samples of the $^{13}C/^{15}N$-labeled GGQ domain of YaeJ, composed of 121 residues, were prepared for structure determination by a cell-free protein expression system. The protein sample had an artificial tag-derived sequence (12 residues, SGPSSGENLYFQ) at the C-terminus, which was derived from the expression vector. NMR resonances were assigned using conventional heteronuclear methods with the $^{13}C/^{15}N$-labeled protein. The assignments of backbone resonances were 95.8% complete for all 121 residues including the tag. Residues with an unassigned backbone were Arg6, His23, His62, Ser66, and Arg78. Tertiary structures were calculated using the CYANA software package (26,27)

based on a total of 2592 NOE-derived distance restraints and 82 backbone torsion angle restraints (Supplementary Table 2). A best-fit superposition of the ensemble of the 20 lowest-energy conformers is shown in Supplementary Figure 2. The root mean square deviation (RMSD) from the mean structure was 0.32 ± 0.04 Å for the backbone (N, C^{α}, C') atoms and 0.74 ± 0.07 Å for all heavy (non-hydrogen) atoms in the well-ordered region of residues 1–22 and 36–99. The statistics of the structures are summarized in Supplementary Table 2.

FIGURE LEGENDS

Supplementary Figure 1. Solution structure of the GGQ domain of the YaeJ protein from *E. coli*. (A) Stereo view illustrating a trace of the backbone atoms of the 20 energy-refined conformers that represent the solution structure (residues 1–109). The GGQ loop is shown in brown. (B) Ribbon diagrams of the structure of the YaeJ GGQ domain. The orientation in the left view is the same as that in (A). On the right, the view is rotated by 180˚ around a vertical axis. The α-helices α1 and α_i are shown in blue and cyan, respectively. The 3_{10} helix, β-strands, and GGQ loop are shown in yellow, light green, and brown, respectively.

Supplementary Figure 2.

(A) Superposition of the structures of the GGQ domains of *E. coli* YaeJ and the YaeJ homolog from *P. syringae* (PDB ID 2JY9), shown in blue and pink, respectively. In the left and middle images, the regions of α1 and α_i are used for determining the optimal superposition. Significant differences in positions of secondary structural elements are indicated by red dotted circles. In the right image, the regions of the β-sheet, composed of β3, β4, and β6, and the β6/α1 loop are used for the superposition, showing significant differences in positions of the N-terminal small β-sheet and α1, indicated by arrows. (B) Superposition of the structures of the GGQ domains of *E. coli* YaeJ in the free form and in the bound form (PDB ID 4DH9), shown in blue and yellow, respectively.

Supplementary Figure 3.

(A) Comparison of surface representations of the electrostatic potential (blue, positive; red, negative) of the GGQ domains of YaeJ and ICT1, calculated by MOLMOL. The

left and right views highlight a surface around Lys53 in α_i of YaeJ and a surface around the corresponding residue, Arg116, in α_i of ICT1, respectively. (B) Roles of conserved residues Lys53 and Arg55 in α_i. Residues that appear to interact with the semi-conserved residues are shown in green. Two putative hydrogen bonds are depicted by red dotted lines.

Supplementary Table 1. List of primers used in this study

Primers used for cloning of the *yaeJ* and *ICT1* genes

	Sequence	Restriction site
yaeJ -Forward	5'-ACTGGCTCATATGattgtgatttcccgacatg-3'	Nde I
yaeJ -Reverse	5'-GATCTCGAGttcccgaccgctgcgcac-3'	Xho I
yaeJ Δ101-140 -Reverse	5'-GATCTCGAGtgttgttaattctttaatca-3'	Xho I
Δ29 ICT1 -Forward	5'-AAGGAGATATACATATGctgcacaagcagaaagacg-3'	Nde I
Δ29 ICT1 -Reverse	5'-GGTGGTGGTGCTCGAGgtccatgtcgaccctcctgcttgtcttta	Xho I

Primers used for mutation of the *yaeJ* gene

Mutants	Forward	Reverse	Method
K53A	5'-ctgccagagtattacGCGgagcgtctg-3'	5'-cagacgctcCGCgtaatactctggcag-3'	Overlap
K53E	5'-ctgccagagtattacGAAgagcgtctg-3'	5'-cagacgctcTTCgtaatactctggcag-3'	Overlap
K53Q	5'-ctgctcgccgccagccatcatttg-3'	5'-acgctcCTGgtaatactctggcaggc-3'	Overlap
R55Q	5'-ctgctcgccgccagccatcatttg-3'	5'-CTGctctttgtaatactctggcaggc-3'	Overlap
L57A	5'-tattacaaagagcgtctgGCCgccgccagccatcat-3'	5'-atgatggctggcggcGGCcagacgctctttgtaata-3'	Overlap
K103A	5'-acagaaaaaGCGgcccgacgaccc-3'	5'-tgttaattctttaatcatagccaccagccg-3'	Inverse
R105A	5'-acagaaaaaaaagccGCAcgacccacg-3'		Inverse
R105Q	5'-caacagaaaaaaaagccCAGcgacccacgcggccc-3'	5'-gggccgcgtgggtcgCTGggcttttttttctgttg-3'	Overlap
R106A	5'-acagaaaaaaaagcccgaGCAcccacg-3'	5'-tgttaattctttaatcatagccaccagccg-3'	Inverse
P107G	5'-gaaaaaaaagcccgacgaGGCacgcggcccacccgt-3'	5'-acgggtgggccgcgtGCCtcgtcgggcttttttttc-3'	Overlap
T108A	5'-GCGcggcccacccgtgcatcgaaagag-3'	5'-gggtcgtcgggcttttttttctgttgttaattc-3'	Inverse
R109A	5'-acgGCGcccacccgtgcatcgaaagag-3'		Inverse
P110A	5'-acgcggGCCacccgtgcatcgaaagag-3'		Inverse
R112A	5'-acgcggcccaccGCGgcatcgaaagag-3'		Inverse
R117A	5'-cacaaaaatcaagcgtgaaggcgatg-3'	5'-ctttcgatgccagcctGGCctctttcgatgcacg-3'	Inverse
R118A	5'-cacaaaaatcaagcgtgaaggcgatg-3'	5'-ctttcgatgccagCGCgcgctctttcgatgcacg-3'	Inverse
R118K	5'-gcatcgaaagagcgcAAActggcatcgaaagcac-3'	5'-gtgctttcgatgccagTTTgcgctctttcgatgc-3'	Overlap
L119A	5'-gaaagagcgcaggGCCgcatcgaaagcacaaaaatc-3'	5'-gatttttgtgctttcgatgcGGCcctgcgctctttc-3'	Overlap
L119Q	5'-gaaagagcgcaggCAGgcatcgaaagcacaaaaatc-3'	5'-gatttttgtgctttcgatgcCTGcctgcgctctttc-3'	Overlap
K122A	5'-gcgcaggctggcatcgGCCgcacaaaaatcaagcg-3'	5'-cgcttgatttttgtgcGGCcgatgccagcctgcgc-3'	Overlap
K122R	5'-gcgcaggctggcatcgCGCgcacaaaaatcaagcg-3'	5'-cgcttgatttttgtgcGCGcgatgccagcctgcgc-3'	Overlap
K125A	5'-GCAtcaagcgtgaaggcgatgcgc-3'	5'-ttgtgctttcgatgccagcctgcg-3'	Inverse
V128A	5'-aaatcaagcGCGaaggcgatgcgc-3'		Inverse
V128Q	5'-aaatcaagcCAGaaggcgatgcgc-3'		Inverse
K129A	5'-caaaaatcaagcgtgGCCgcgatgcgcggcaaag-3'	5'-ctttgccgcgcatcgcGGCcacgcttgatttttg-3'	Overlap
K129R	5'-caaaaatcaagcgtgCGCgcgatgcgcggcaaag-3'	5'-ctttgccgcgcatcgcGCGcacgcttgatttttg-3'	Overlap
R132A	5'-tgaaggcgatgGCCggcaaagtgcg-3'	5'-cgcactttgccGGCcatcgccttca-3'	Overlap
R132K	5'-caagcgtgaaggcgatgAAAggcaaagtgcgcagc-3'	5'-gctgcgcactttgccTTTcatcgccttcacgcttg-3'	Overlap
R117A/R118A	5'-cacaaaaatcaagcgtgaaggcgatg-3'	5'-ctttcgatgccagCGCGGCctctttcgatgcacg-3'	Inverse
K122A/K129A for R132A mutant	5'-GCGgcacaaaaatcaagcgtgGCCgcgatg-3'	5'-cgatgccagcctgcgctctttcgatgc-3'	Inverse
Δ100	5'-gaaaaaaaagcccgacgacccacg-3'	5'-tgttaattctttaatcatagccaccagccg-3'	Inverse
Δ100-101	5'-aaaaaagcccgacgacccacg-3'		Inverse
Δ100-102	5'-aaagcccgacgacccacgcgg-3'		Inverse

Primers used for mutation of the *ICT1* gene

Mutants	Forward	Reverse	Method
ICT1 R187A	5'-GCCctgagacaaaagagaattc-3'	5'-ttcccgattcatgttttctatcctg-3'	Inverse
ICT1 K191A	5'-aggctgagacaaGCCagaattc-3'		Inverse
ICT1 K198A	5'-GCCacaagcaggagggtcgacatgg-3'	5'-tacagcagaatgaattctcttttg-3'	Inverse
ICT1 R201A	5'-aagacaagcGCCagggtcgacatgg-3'		Inverse
ICT1 Δ171-173	5'-aaacttcatagaatcaggatag-3'	5'-ttttgttggctccttcggtgtc-3'	Inverse

Supplementary Table 2. Summary of conformational constraints and statistics of the NMR solution structure of the YaeJ protein from *E. coli*.

NOE upper distance restraints	
Short-range ($\|i - j\| \leq 1$)	1217
Medium-range ($1 < \|i - j\| < 5$)	521
Long-range ($\|i - j\| \geq 5$)	859
Total	2592
Dihedral angle restraints (ϕ and ψ)	82
CYANA target function value ($Å^2$)	1.79 ± 0.29
Number of restraint violations	
Distance restraint violations (> 0.1 Å)	0
Dihedral angle restraint violations (> 2.5˚)	0
AMBER energies (kcal/mol)	
Total	–4169 ± 98
van der Waals	–289 ± 15
Electrostatic	–4762 ± 97
Ramachandran plot (%)	
Residues in most favored regions	79.1
Residues in additional allowed regions	19.5
Residues in generously allowed regions	1.3
Residues in disallowed regions	0.1
RMSD deviation from the averaged coordinates (Å) (res. 1-20, 36-99)	
Backbone atoms	0.32 ± 0.04
Heavy atom	0.74 ± 0.07

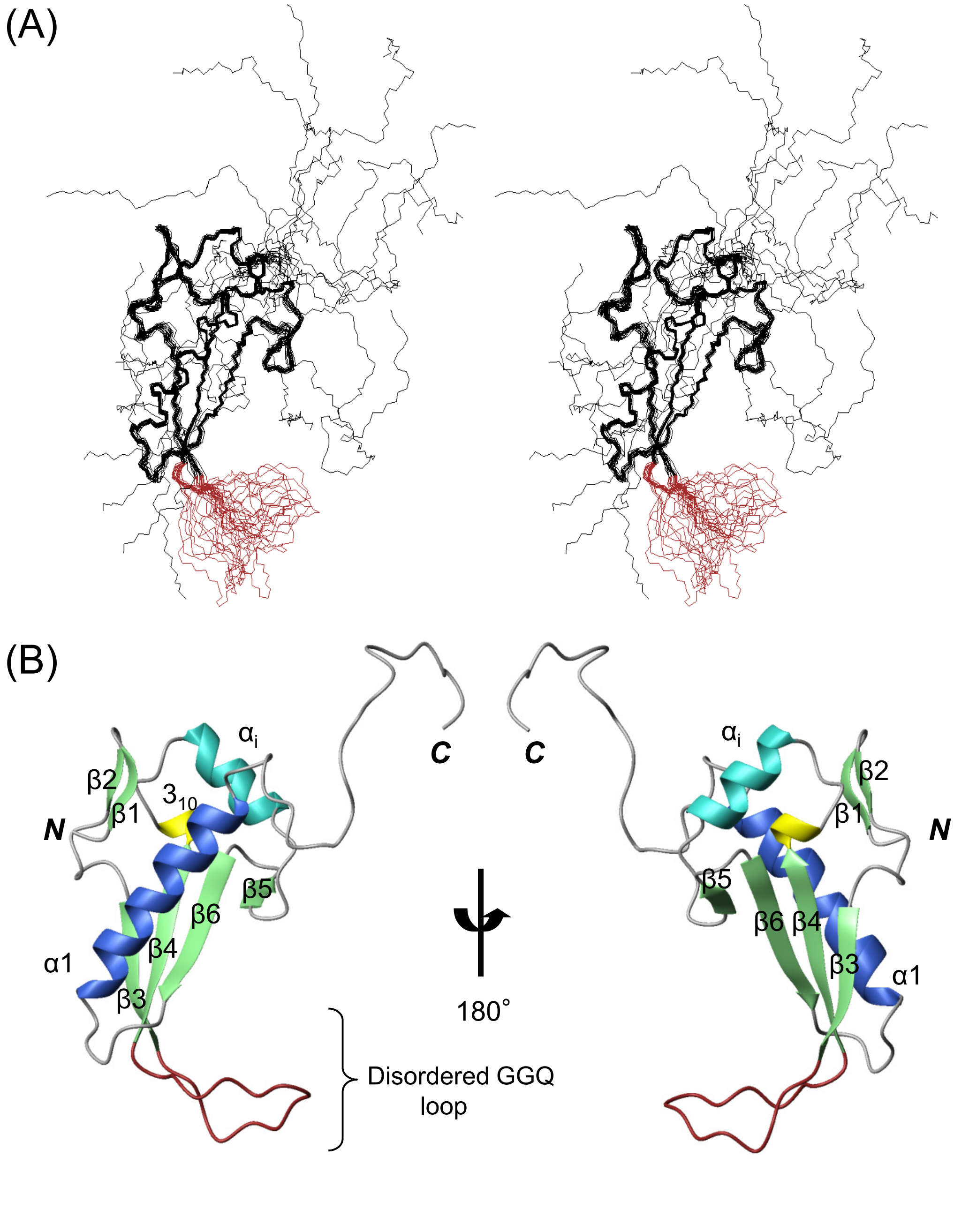

Supplementary Fig. 1

(A) *E. coli* vs. *P. syringae*

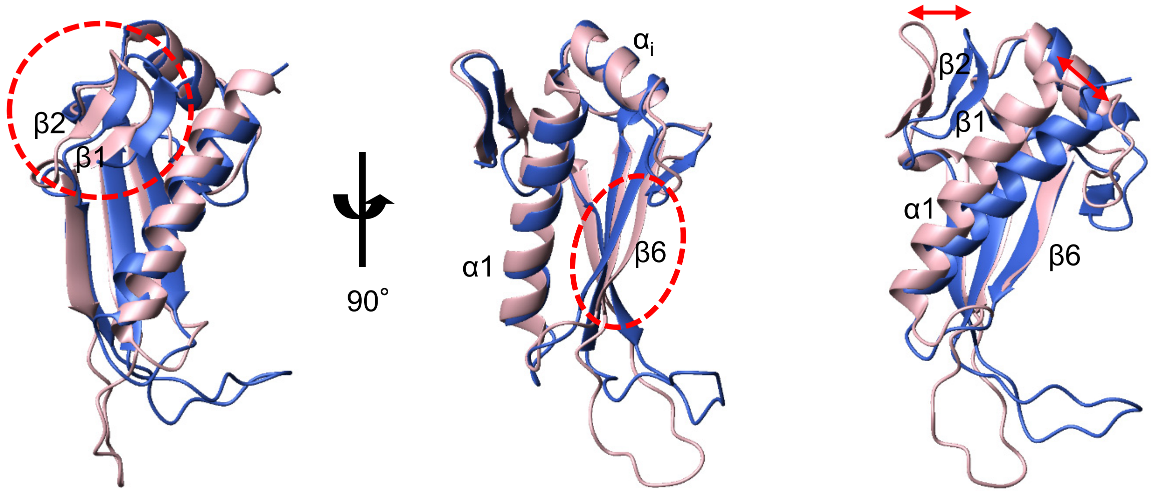

(B) Free form vs. bound form

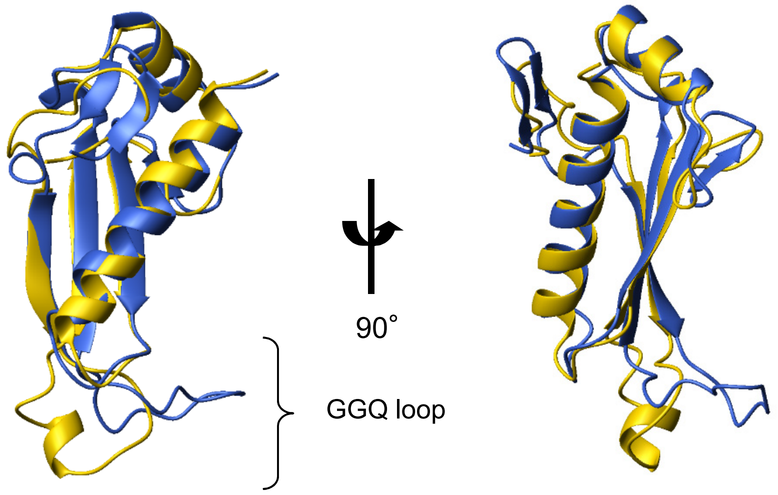

Supplementary Fig. 2

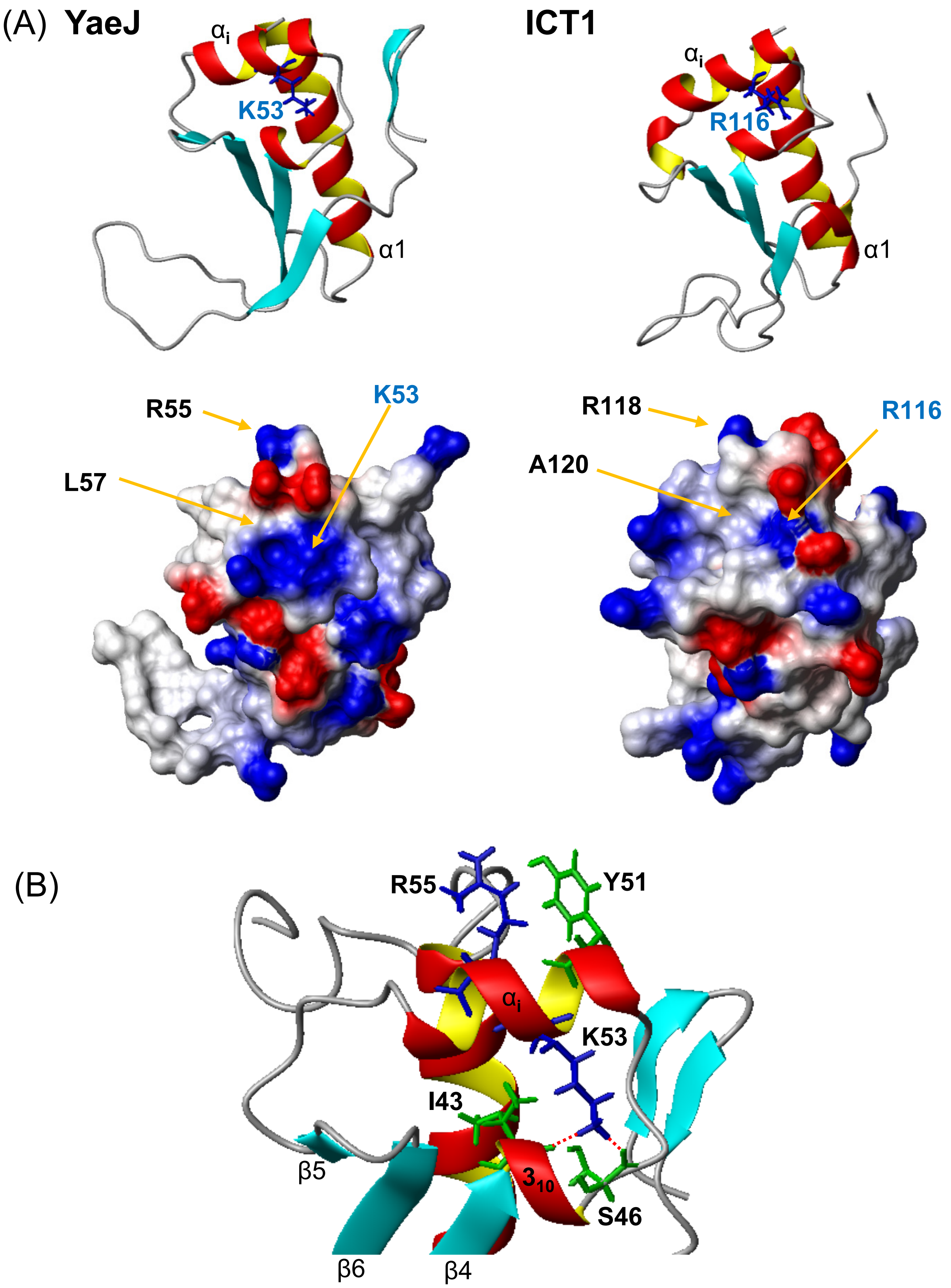

Supplementary Fig. 3
